# Embracing complexity in primary care

**DOI:** 10.4102/safp.v64i1.5519

**Published:** 2022-04-19

**Authors:** Klaus B. von Pressentin

**Affiliations:** 1 Division of Family Medicine, School of Public Health and Family Medicine, Faculty of Health Sciences, University of Cape Town, Cape Town, South Africa

During a Sunday morning cycle at the foot of the majestic Table Mountain, I was struck by how many routes there are to consider, including man-made and natural trails, routes to explore by mountain bike or on foot and routes to enjoy alone, in pairs or groups. As I cycled uphill, I reflected on how this network of interconnecting roads and destinations could symbolise the complexities we so often encounter in our daily primary care activities. In this editorial, I highlight a few examples that require us to work with polarities and require moving from a binary view to a perspective that seeks to understand the granularity within the spectrum, as well as across multiple dimensions.

The context of primary care is often dichotomised into either urban versus rural health care, public versus private sector, community health centre versus district hospital, city clinic versus general practitioner practice, or facility-based versus community-based health care. In the consultation space, the patient could be seen over time by the same primary care provider versus different members of the primary care team at each episode or contact with the healthcare system. Care provided may be seen as either reactive versus proactive/preventative or curative versus palliative. Our patients are juggling a lived experience informed by multimorbidity coupled with a range of social determinants of health, whilst our health system and clinical guidelines seem to prefer single-disease, linear approaches to evidence-based care. The spectrum of care providers may be viewed as a horizontal line, with providers graded according to their credentials and experience (including community health worker with certificate vs. medical doctor with a degree, medical intern vs. consultant) and whether their disciplinary identity is generalist or specialist. Generalist care is often juxtaposed with specialist care by the public, health professionals, policymakers and the media, with the former often viewed as simple and common versus the latter as expert and sought-after. The underpinning philosophies of primary care may also be regarded from the perspectives of the different disciplines that share this space of practice and scholarly discourse (including medical vs. non-medical), by World Bank country classifications (high- vs. middle- vs. low-income), by disciplinary epistemology (person-centred vs. community-orientated, holistic vs. organ-specific, science vs humanities) and by Western versus traditional worldviews (including allopathy vs. homeopathy).

The limitations of having a dichotomised view become obvious in our daily interactions with our patients, students and colleagues. We become frustrated and burnt out when our patients are not able to change their unhealthy behaviours because of the constraints of their social environment, including the lack of access to basic needs, education and employment. We become despondent when our students choose a career outside the primary care context and become part of the brain drain rate and depletion of skilled human resources in our home country. We revert to our nationalistic approach of promoting the interests of our own discipline and health worker category above those of the other members of the healthcare team, especially when faced with and threatened by conflicting policy directions regarding human resources and health workforce priorities at institutional, national and international levels.

What will help us to break away from this pattern of thinking and to become more appreciative of other and new perspectives? The Leadership Maturity Framework provides one possible solution.^1,2^ This framework is based on Jane Loevinger’s ego development theory with further enhancement by Susanne Cook-Greuter’s research and describes nine ways of adult meaning making. Polarities are generated with language (as I have illustrated by the terminology used in the paragraphs above). These polarities are unavoidable, interdependent and ongoing. We should strive to create caring, holding spaces in which to embrace complexity, including spaces that are inclusive yet respect disruptive conversations and embrace diverse views and interdisciplinary ‘entanglement’.^3^ We hope that the South African Academy of Family Physicians, its interest groups, its meetings and conferences, and its journal will represent such spaces for the Southern African primary care community. Recently, we have appointed additional assistant editors and a new editorial board for the South African Family Practice journal, as well as updated the editorial board’s terms of reference.^4^ These actions confirm the editorial team’s commitment to this brief.

Adam Grant talks about embracing ‘confident humility’ in his book, *Think Again*.^5^ This ‘sweet spot of confidence’ lies mid-way between the confidence in your ability to achieve a future goal and your ability to question your own competence, thereby maintaining the humility to question whether you have the right tools or solutions in the present. Like navigating the many roads and destinations available on Table Mountain, it is helpful to reflect on the fitness level of your ability to deal with life’s complexities and to know when to reach out to fellow road users for directions. We are all aspiring lifelong learners and those who cross our path may have something of value for us to learn from, especially those who use a different mode or speed of travel. The ability to rethink our approach to polarity and to embrace a ‘confident humility’ approach to complexity thinking will help us to view challenges as opportunities for adaptation and innovation whilst allowing us to engage meaningfully and comfortably during periods of transition and transformation.^6,7^

Best wishes,

Klaus B. von Pressentin

Editor-in-Chief

Twitter: @klausvon; @SAFPjournal
